# Artemether-Lumefantrine Versus Chloroquine for the Treatment of Uncomplicated *Plasmodium knowlesi* Malaria: An Open-Label Randomized Controlled Trial CAN KNOW

**DOI:** 10.1093/cid/cix779

**Published:** 2017-10-23

**Authors:** Matthew J Grigg, Timothy William, Bridget E Barber, Giri S Rajahram, Jayaram Menon, Emma Schimann, Christopher S Wilkes, Kaajal Patel, Arjun Chandna, Ric N Price, Tsin W Yeo, Nicholas M Anstey

**Affiliations:** 1 Global and Tropical Health Division, Menzies School of Health Research, Darwin, Northern Territory, Australia; 2 Infectious Diseases Society, Sabah-Menzies School of Health Research Clinical Research Unit, Kota Kinabalu; 3 Clinical Research Centre, Queen Elizabeth Hospital, Kota Kinabalu, Malaysia; 4 Jesselton Medical Centre, Kota Kinabalu, Malaysia; 5 Sabah Department of Health, Kota Kinabalu, Malaysia; 6 Centre for Tropical Medicine and Global Health, Nuffield Department of Clinical Medicine, University of Oxford, United Kingdom; 7 Lee Kong Chian School of Medicine, Nanyang Technological University, Singapore; 8 Division of Medicine, Royal Darwin Hospital, Darwin, Northern Territory, Australia

**Keywords:** *Plasmodium knowlesi*, malaria, randomized controlled trial, artemether-lumefantrine, chloroquine

## Abstract

**Background:**

*Plasmodium knowlesi* is reported increasingly across Southeast Asia and is the most common cause of malaria in Malaysia. No randomized trials have assessed the comparative efficacy of artemether-lumefantrine (AL) for knowlesi malaria.

**Methods:**

A randomized controlled trial was conducted in 3 district hospitals in Sabah, Malaysia to compare the efficacy of AL against chloroquine (CQ) for uncomplicated knowlesi malaria. Participants were included if they weighed >10 kg, had a parasitemia count <20000/μL, and had a negative rapid diagnostic test result for *Plasmodium falciparum* histidine-rich protein 2. Diagnosis was confirmed by means of polymerase chain reaction. Patients were block randomized to AL (total target dose, 12 mg/kg for artemether and 60 mg/kg for lumefantrine) or CQ (25 mg/kg). The primary outcome was parasite clearance at 24 hours in a modified intention-to-treat analysis.

**Results:**

From November 2014 to January 2016, a total of 123 patients (including 18 children) were enrolled. At 24 hours after treatment 76% of patients administered AL (95% confidence interval [CI], 63%–86%; 44 of 58) were aparasitemic, compared with 60% administered CQ (47%–72%; 39 of 65; risk ratio, 1.3 [95% CI, 1.0–1.6]; *P* = .06). Overall parasite clearance was shorter after AL than after CQ (median, 18 vs 24 hours, respectively; *P* = .02), with all patients aparasitemic by 48 hours. By day 42 there were no treatment failures. The risk of anemia during follow-up was similar between arms. Patients treated with AL would require lower bed occupancy than those treated with CQ (2414 vs 2800 days per 1000 patients; incidence rate ratio, 0.86 [95% CI, .82–.91]; *P* < .001). There were no serious adverse events.

**Conclusions:**

AL is highly efficacious for treating uncomplicated knowlesi malaria; its excellent tolerability and rapid therapeutic response allow earlier hospital discharge, and support its use as a first-line artemisinin-combination treatment policy for all *Plasmodium* species in Malaysia.

**Clinical trials registration:**

NCT02001012.

## Teaser

AL is highly efficacious for treating uncomplicated *Plasmodium knowlesi* malaria; its excellent tolerability and rapid therapeutic response allowed earlier hospital discharge, and support its use as a first-line artemisinin-combination therapy treatment policy for all *Plasmodium* species in Malaysia.

The simian parasite *Plasmodium knowlesi* is reported increasingly in humans infected across Southeast Asia [1, 2] and is now the most common cause of malaria in Malaysia [3–5] and areas of western Indonesia [6, 7]. Accurate reporting remains difficult in endemic areas owing to the inability of routine microscopy to differentiate *P. knowlesi* from *Plasmodium malariae* or the early ring stage of *Plasmodium falciparum*, with misidentification as *Plasmodium vivax* also occurring [2, 8, 9]. Misidentification of *P. knowlesi* has been associated with failure to recognize severe disease and death [10–12], and inappropriate treatment with chloroquine (CQ) of resistant *P. falciparum* or *P. vivax* infections. Rapid diagnostic tests lack sensitivity and specificity for *P. knowlesi*, and cannot be used to guide prompt and effective treatment [13–15].

The predominant zoonotic reservoir of *P. knowlesi* reduces the antimalarial drug selection pressure, with *P. knowlesi* shown to respond rapidly to numerous antimalarials, including artemisinin-combination therapy (ACT) [16, 17] and CQ [16–18]. To date, clinical failure after the treatment of uncomplicated knowlesi malaria has not been reported [19], with the high efficacy of the main antimalarial options confirmed by an in vitro drug susceptibility study [20]. In the only randomized controlled trial conducted for the treatment of uncomplicated knowlesi malaria, artesunate-mefloquine (ASMQ), compared with CQ, resulted in faster parasite and fever clearance, lower bed occupancy, and reduced risk of anemia [16]. The current World Health Organization (WHO) 2015 malaria treatment guidelines recommend either ACT or CQ for uncomplicated *P. knowlesi* infection in CQ-susceptible areas or first-line ACT in areas with confirmed CQ-resistant *P. vivax* [21], as found in Malaysia [22].

Artemether-lumefantrine (AL) is widely used as the first-line ACT for malaria in endemic countries, including Malaysia [23, 24]. AL is better tolerated than ASMQ, which causes gastrointestinal disturbance and rare but serious neuropsychiatric adverse effects [16, 25]. However, systematic evaluation of AL and comparison with CQ for the treatment of knowlesi malaria is warranted, given the significant differences in the pharmacokinetics between AL and ASMQ that may affect clinical outcomes [21]. The aim of the current study was to compare the clinical efficacy of AL with CQ, in order to optimize the treatment of uncomplicated *P. knowlesi* malaria in both adults and children in areas where either AL or CQ may be currently used.

## METHODS

### Trial Design

A 2-arm, randomized, open-label trial conducted at 3 sites in Sabah, Malaysia: Kudat, Kota Marudu, and Pitas district hospitals, where *P. knowlesi* is the most common cause of malaria [26, 27]. Participants included patients with uncomplicated *P. knowlesi* malaria presenting to the study hospital sites and meeting the following inclusion criteria: ≥1 year of age, weight ≥10 kg, microscopic diagnosis of *P. knowlesi* monoinfection, negative results of a rapid diagnostic test for *P. falciparum* malaria (histidine-rich protein 2), fever (temperature ≥37.5°C) or history of fever in the last 48 hours, and provision of written informed consent. Patients with clinical or laboratory criteria for severe malaria or warning signs according to WHO 2014 criteria [28], parasitemia counts >20000/μL [17], pregnancy or lactation, known hypersensitivity or allergy to study drugs, serious underlying disease (cardiac, renal or hepatic), or any antimalarial use in the previous 2 months were excluded. This study was approved by the relevant human medical research ethics committees of Malaysia and Menzies School of Health Research, Australia

### Interventions

AL (Riamet; Novartis), was given as a fixed-dose combination of artemether (20 mg) and lumefantrine (120 mg). Doses were administered at enrolment and at 8, 24, 36, 48, and 60 hours, with target total doses of 12 mg/kg for artemether and 60 mg/kg for lumefantrine. Chloroquine phosphate (Axcel; Kotra Pharma), consisting of 155-mg base tablets, was administered at enrolment and at 6, 24, and 48 hours, with a target total dose of 25 mg/kg. Dosages of AL and CQ were based on weight, consistent with current WHO [21] and Malaysian Ministry of Health treatment guidelines [24]. Administration of all doses was supervised by a member of the study team, and all patients were observed for 1 hour after treatment, with readministration of the full dose if vomiting occurred. Coadministration of fatty foods with AL was recommended but not supervised, according to local practice.

### Outcomes

The primary end point was the difference between the 2 treatment arms in the proportion of patients with negative microscopic results for *P. knowlesi* asexual parasites at 24 hours. Secondary end points included microscopic parasite clearance as measured by time to the first of 2 negative blood films; the times to 50%, 90%, and 99% reduction in baseline parasite count in those with parasitemia counts >1000/μL [29]; and the proportion of patients aparasitemic at 48 and 72 hours. Other a priori secondary end points included parasitological cure at days 28 and 42 [21], risk of anemia at day 28, fractional fall in hemoglobin at day 3, the nadir of the hemoglobin concentration, risk of adverse and serious adverse events and relationship to study drugs, and the predicted length of hospital inpatient stay. Anemia was defined according to the WHO age-based criteria [30].

### Power Calculation

A sample size of 58 participants in each arm would have 90% power to falsify the null hypothesis (no difference in parasite clearance between AL and CQ at 24 hours), with a significance level (α) of .05, assuming that 45% of study participants treated with CQ and 16% of those treated with ACT would remain parasitemic 24 hours after the start of treatment [16].

### Randomization

Patient allocation codes were computer generated in blocks of 20 for each drug arm using Stata software (version 12) before the start of the study. Treatment allocation was provided in a sealed opaque envelope opened by a study nurse once the participant met all the study enrollment criteria and informed consent had been signed.

### Blinding

The primary study end point was determined by microscopists who were blinded to treatment allocation. Research blood slides were labeled only with the study code, which was nonidentifiable for both demographic data and drug allocation. Staff conducting all biochemical and polymerase chain reaction (PCR) assays were blinded to results at microscopy and treatment arm.

### Study Procedures

Venous blood was taken before enrollment for hematology, biochemistry, rapid diagnostic tests, and delayed species PCR, as described elsewhere [31, 32]. All patients had symptoms recorded daily, and 6-hourly finger-prick blood sampling to assess microscopic parasite clearance. Vital signs were documented, and venous blood obtained for hematological investigations, daily during admission and at each follow-up visit at days 7, 14, 28, and 42 after initial treatment. Patients were discharged from the hospital once they had completed their study drug administration and 2 consecutive blood slides were negative for asexual parasites.

### Statistical Methods

Data were double entered by separate individuals into Epidata software (version 3.1), and analyzed using Stata software (version 12). Primary analysis of parasite clearance at 24 hours was by modified intention to treat. Intergroup differences were compared using the Student *t* test or the Wilcoxon-Mann-Whitney test for continuous variables and χ^2^ or the Fisher exact test for categorical variables. Microscopic asexual parasite and gametocyte counts were calculated using thick blood smears [16]. Best-fit linear or tobit polynomial regression models were used to estimate the curve of natural logarithm (log_e_) parasite counts versus time using the WorldWide Antimalarial Resistance Network (WWARN) parasite clearance method [29]. Treatment outcomes were evaluated by means of Kaplan Meier survival analysis. Meta-analysis of the weighted difference in risk of the study primary end point for AL versus CQ was compared with findings in a previous randomized trial comparing ASMQ and CQ [16], using a Mantel-Haenszel fixed-effects model of individual patient data, including a test of study heterogeneity (*I*^2^).

## RESULTS

**Figure 1.**
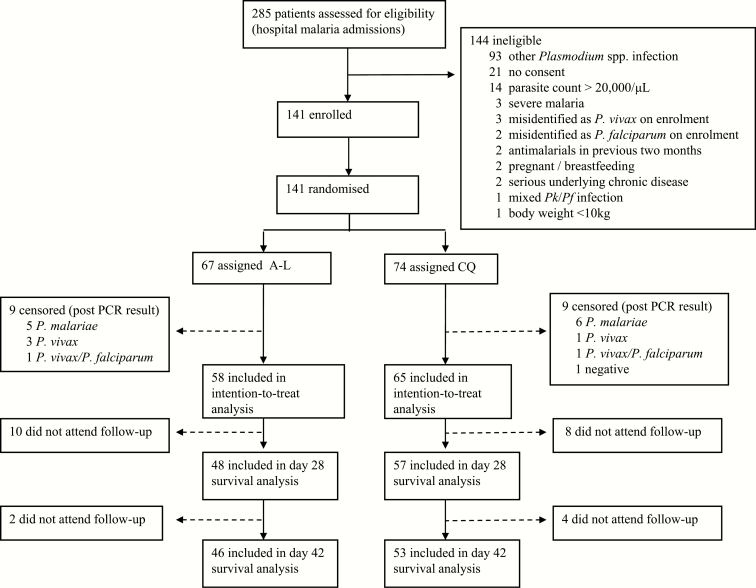
Enrollment flowchart. Abbreviations: AL, artemether-lumefantrine; CQ, chloroquine; *P., Plasmodium*; PCR, polymerase chain reaction.

Of the 285 malaria admissions at all study sites over the enrollment period (21 November 2014 to 6 January 2016), 186 patients (65%) had *P. knowlesi* monoinfection microscopically diagnosed, with 141 (76%) enrolled in the study (Figure 1). Seventeen patients initially randomized were excluded from the analysis owing to a PCR diagnosis of *P. vivax*, *P. malariae,* mixed *P. vivax*/*P. falciparum,* or a negative result, leaving 58 patients in the AL arm and 65 in the CQ arm for the primary analysis.

There were no differences in baseline demographics of the patients enrolled between study arms (Table 1). The median age of patients was 31 (range, 4–79) years with 15% (18 of 123) children aged ≤12 years. The geometric mean parasitemia count at presentation was 1437/μL (range, 57–44744/μL) in the AL arm and 1485/μL (89–32660/μL) in the CQ arm, of whom 7 (6%) had a parasitemia count <100/µL. The subsequent cross-check of enrollment hospital slides by research microscopists identified 8 patients in the modified intention-to-treat analysis with parasitemia >20000/μL; 4 of these patients had been randomized to each treatment arm. The mean parasitemia counts at presentation were similar between treatment arms for children. In total 83% of patients (48 of 58) in the AL arm and 88% (57 of 65) in the CQ arm completed day 28 follow-up and were included in the secondary survival analysis of the treatment outcome. No patients in either treatment arm had either early or late treatment failure.

**Table 1.** Demographic and Baseline Characteristics

Variable	AL Arm (n = 58)	CQ Arm (n = 65)
Age, median (IQR) [range], y	30 (17–45) [7–79]	31 (21–43) [4–75]
Age ≤12 y, No. (%)	7 (12.1)	5 (7.7)
Male sex, No. (%)	49 (84.5)	46 (70.8)
Body weight, median (IQR), kg	55 (50–65)	57 (47–70)
Fever (temperature ≥37.5°C) at admission, No. (%)	22 (37.9)	33 (50.8)
Duration of fever history, median (IQR) [range], d	4 (3–5) [1–10]	5 (4–7) [2–14]
Parasite count, geometric mean (95% CI) [range], parasites/μL		
All patients	1437 (864–2390) [57–44744]	1485 (1006–2193) [89–32660]
Children (age ≤12 y)	984 (212–4565) [183–9229]	3443 (408–29044) [274–32320]
Synchronous infections, No. (%)^a^		
Rings	16 (27.6)	18 (27.7)
Trophozoites	42 (72.4)	47 (72.3)
Gametocytes present. No. (%)	15 (25.9)	17 (26.2)
Hemoglobin median (IQR) [range], g/dL	13.4 (11.9–14.7) [7.5–17.0]	13.1 (11.9–14.2) [7.1–15.3]
Creatinine, median (IQR), mmol/L	85 (55–97)	86 (66–97)
Total dose administration		
Target total dose, mg/kg	Artemether: 12; lumefantrine: 60	25
Median (IQR) [range], mg/kg	Artemether: 8.7 (7.0–9.6) [4.9–13.3]; lumefantrine: 52.0 (41.0–57.6) [29.2–80.0]	26.3 (22.0–31.0) [17.8–44.3]
Body weight ≤35 kg		
No. (%)	7 (12.1)	7 (10.8)
Median (IQR) [range], mg/kg	Artemether: 11.7 (10.2–13.1) [9.8–13.3]; lumefantrine: 69.9 (61.1–78.5) [58.5–80.0]	24.8 (24.1–38.8) [23.3–44.3]
Body weight >35 kg		
No. (%)	51 (87.9)	58 (89.2)
Median (IQR) [range], mg/kg	Artemether: 8.0 (6.8–9.2) [4.9–13.3]; lumefantrine: 48.0 (40.9–55.3) [29.2–79.6]	26.6 (21.8–31.0) [17.8–39.7]

Abbreviations: AL, artemether-lumefantrine; CI, confidence interval; CQ, chloroquine; IQR, interquartile range.

^a^Defined as a single life-cycle stage comprising >70% of all asexual parasites

The proportion of patients aparasitemic at 24 hours was 76% (44 of 58) in the AL arm and 60% (39 of 65) in the CQ arm (difference in proportion, 16%; 95% confidence interval [CI], 0%–33%; *P* = .06). OF patients with parasitemia counts >1000/μL at enrollment [29], 22 of 35 (63%) treated with AL and 15 of 38 (40%) treated with CQ were aparasitemic at 24 hours (difference in proportion, 23%; 95% CI, 0%–46%; *P* = .047; Table 2). In children ≤12 years old, 86% (6 of 7) treated with AL were aparasitemic at 24 hours, compared with 20% (1 of 5) in the CQ arm (difference in proportion, 66%; 95% CI, 13%–100%; *P* = .02. All patients in the AL arm were aparasitemic) by 42 hours, compared with 48 hours for the CQ arm. Overall, the median parasite clearance time was 18 hours (range, 6–42 hours) after AL versus 24 hours (12–48 hours) after CQ (*P* = .02); the corresponding slopes of the log-normalized clearance curves were 0.27 and 0.22, respectively (*P* < .002). Of patients with parasitemia counts >20000/μL, parasite clearance times ranged from 24 to 36 hours for AL, and from 30–42 hours for CQ (*P* = .16). No patients had microscopic gametocytemia at any time point during follow-up, and there was no statistically significant difference in fever clearance time between treatment arms (Table 2).

**Table 2.** Treatment Outcomes; Parasite and Fever Clearance

Variable	AL Arm (n = 58)	CQ Arm (n = 65)	*P* Value
Adequate parasitological and clinical response, No./total (%)^a^			
Day 28	48/48 (100)	57/57 (100)	…
Day 42	46/46 (100)	53/53 (100)	…
Parasitological response: any parasitemia			
24 h			
Negative results, No. (%; 95% CI)	44 (76; 63–86)	39 (60; 47–72)	.06
Difference in risk (95% CI), %	16 (0–32)		…
Risk ratio (95% CI)	1.3 (1.0–1.6)		…
48 h			
Negative results, No. (%; 95% CI)	58 (100; 93.8–100)	65 (100; 94.5–100)	…
PCT, median (IQR) [range], h	18 (18–24) [6–42]	24 (18–30) [12–48]	.02^b^
Parasitological response: parasitemia >1000/μL^a^	n = 35 (60%)	n = 38 (58%)	
24 h			
Negative results, No. (%; 95% CI)	22 (63; 46–80)	15 (40; 23–56)	.047^b^
Difference in risk (95% CI), %	23 (0–46)		…
Risk ratio (95% CI)	1.6 (1.0–2.5)		…
48 h			
Negative results, No. (%; 95% CI)	35 (100; 90.0–100)	38 (100; 90.7–100)	…
PCT, median (IQR) [range], h	24 (18–30) [12–42]	30 (24–36) [12–48]	.02^b^
Slope of curve for log_10_-normalized parasite clearance mean (95% CI), *k*^c^	0.27 (.25–.29)	0.22 (.20–.24)	.002^b^
Lag phase present, No. (%)	14 (40.0)	13 (34.2)	.61
Lag phase duration, mean (95% CI), h	2.9 (1.6–4.3)	2.5 (1.3–3.7)	.81
PCT, mean (95% CI), h			
50% PCT ^d^	7.2 (5.6–8.9)	8.2 (6.4–10.1)	.51
90% PCT	13.7 (11.8–15.7)	15.6 (13.6–17.7)	.14
99% PCT	23.1 (20.3–25.8)	26.9 (24.2–29.6)	.03^b^
Fever clearance time, median (IQR) [range], h	18 (12–24) [6–60]	12 (12–30) [6–48]	.87

Abbreviations: AL, artemether-lumefantrine; CI, confidence interval; CQ, chloroquine; IQR, interquartile range; PCT, parasite clearance time.

^a^Adequate parasitological and clinical response is defined elsewhere [21].

^b^Significant difference (*P* < .05).

^c^Including only those with parasite counts >1000/μL according to the WorldWide Antimalarial Resistance Network (WWARN) parasite clearance method [29].

^d^Reduction in parasite count by 50%.

The overall risk of anemia throughout the 28-day follow-up was 81% (46 of 57) in the CQ arm, compared with 67% (32 of 48) in the AL arm (*P* = .10; Table 3**).** Of the patients without anemia at baseline, 65% (28 of 43) in the CQ arm became anemic by day 28, compared with 56% (23 of 41) in the AL arm (*P* = .68).

**Table 3.** Hematological Outcomes

Variable	AL Arm (n = 58)	CQ Arm (n = 65)	*P* Value
Fractional fall in hemoglobin at d 3, mean (95% CI), %	10.1 (8.0–12.1)	12.9 (10.8–15.0)	.055
Hemoglobin nadir, median (IQR) [range], g/dL	11.9 (10.5–12.7) [7.1–14.6]	11.1 (9.9–12.1) [7.0–14.1]	.20
Time to hemoglobin nadir, median (IQR} [range], d	3 (2–7) [1–28)]	2 (2–3) [1–28]	.008^a^
Prevalence of anemia, % (95% CI) [No./total]^b^			
Baseline	29.3 (17.2–41.4) [17/58]	33.8 (22.0–45.7) [22/65]	.59
At d 28	29.2 (17.0–44.0) [14/48]	35.1 (22.9–48.9) [20/57]	.52
Throughout 28-d follow-up	66.7 (51.6–79.6) [32/48]	80.7 (68.1–90.0) [46/57]	.10

Abbreviations: AL, artemether-lumefantrine; CI, confidence interval; CQ, chloroquine; IQR, interquartile range.

^a^Significant difference (*P* < .05).

^b^Anemia defined according to World Health Organization age-based criteria [30]

The actual durations of hospital stays were similar between treatment arms (median, 3 days; range, 1–5 days). When applying the national hospital discharge policy of 2 negative blood slides on consecutive days with medication to be completed after discharge, the predicted bed occupancy was 2414 days per 1000 patients treated with AL versus 2800 days per 1000 patients treated with CQ (incidence rate ratio, 0.86; 95% CI, .82–.91; *P* < .001).

Mild dyspnea was reported more frequently in patients treated with AL than in those treated with CQ (7% vs 0%, respectively; *P* = .03), although there were no differences in the reporting of cough, respiratory rate, or oxygen saturation. No other differences in adverse events were documented (Table 4). The most common adverse events were rash or itch, reported in 8% of patients, and dizziness, cough, or vomiting in 6%. Only a single patient in the AL group required readministration of the first dose of study medication within 1 hour after vomiting. No patients required rescue treatment or had a serious adverse event.

**Table 4.** Adverse Events

Adverse Event	Patients, No. (%)		*P* Value
	AL Arm	CQ Arm	
Gastrointestinal			
Vomiting	2 (3)	5 (8)	.31
Abdominal pain	3 (5)	3 (5)	.89
Diarrhea	2 (3)	2 (3)	.91
Neurological			
Dizziness	3 (5)	4 (6)	.82
Headache	2 (3)	3 (5)	.74
Vision/hearing	2 (3)	1 (2)	.49
Skin: rash or itch	7 (12)	3 (5)	.13
Respiratory			
Cough	3 (5)	4 (6)	.82
Shortness of breath	4 (7)	0 (0)	.03^a^
Retreatment due to vomiting <1 h after dosing	1 (2)	0 (0)	.29
Rescue treatment	0	0	…

Abbreviations: AL, artemether-lumefantrine; CQ, chloroquine.

^a^Significant difference (*P* < .05).

**Figure 2.**
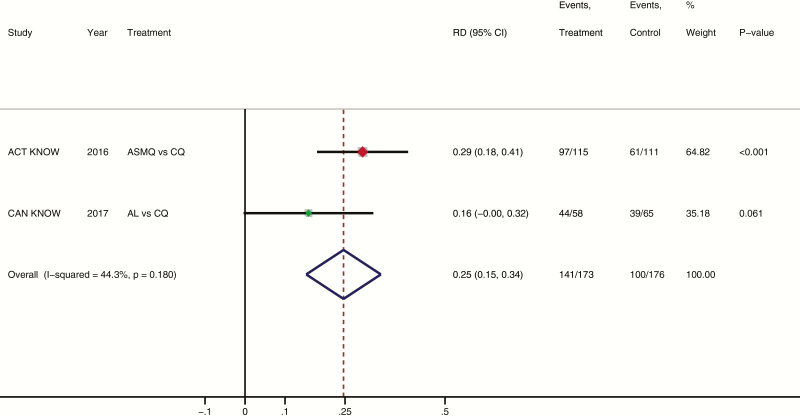
Meta-analysis of artemisinin-combination therapy (ACT) versus chloroquine (CQ) for treatment of *Plasmodium knowlesi* malaria, showing difference in proportional parasite clearance at 24 hours. The red and green squares are the relative weight given to each study in the meta-analysis (ie, 65 and 35%, respectively) based on the number of participants in the 2 studies (226 vs 123). The proportional difference in risk of parasite clearance at 24 hours between treatment arms. Abbreviations: ACT, artemisinin-combination therapy; AL, artemether-lumefantrine; ASMQ, artesunate-mefloquine; CI, confidence interval; CQ, chloroquine; RD, risk difference.

A meta-analysis using individual patient data for the difference in proportional parasite clearance at 24 hours between AL and CQ arms (16%) and a previous trial of ASMQ versus CQ (29%) [16] demonstrated comparable primary outcomes (*I*^2^ = 44%; *P* = .18; Figure 2). The combined weighted difference in parasite clearance at 24 hours was 25% (95% CI, 15%–34%) in those treated with either ACT compared with CQ (*P* < .001). Comparing direct treatment outcomes between AL and ASMQ, there was no statistically significant difference in proportions negative for parasites at 24 hours (76% [44 of 58] vs 84% [97 of 115], respectively), bed occupancy (2414 vs 2426 days per 1000 patients), or risk of anemia at 28 days (29% vs 32%).

## DISCUSSION

AL and CQ were highly effective for treating uncomplicated knowlesi malaria, with both drugs well tolerated, all patients becoming aparasitemic within 48 hours, and no treatment failures by day 42 of follow-up. However, AL demonstrated faster parasite clearance and earlier predicted hospital discharge than CQ, with improved potential health cost benefits. The earlier parasite clearance for AL compared with CQ is consistent with a previous trial of ASMQ versus CQ for uncomplicated knowlesi malaria [16].

The difference in parasite clearance between AL and CQ was more pronounced in those presenting with higher parasite counts >1000/μL, although this may be due in part to methodologic difficulties in accurately evaluating parasite clearance at lower levels [29]. Routine screening hospital microscopy was demonstrated to underestimate parasite counts in a number of patients, including those with counts above the 20000/μL inclusion threshold. Microscopic underestimation of *P. knowlesi* parasite counts and inappropriate initial treatment with oral rather than intravenous artesunate for severe disease has resulted in case fatalities [12]. However, oral ACT may minimize the risk of worsening complications for unrecognized moderate or severe disease compared with drugs with slower parasite clearance, such as CQ. Parasitemia is a known risk factor for severe knowlesi malaria [17, 33, 34], with parasitemia counts >20000/μL resulting in an 11-fold increased risk of severe disease [17]. The safety of oral therapy for uncomplicated disease with parasite counts above this threshold has not been systematically evaluated, although both ACT and CQ were successfully used to treat a small number of patients with parasite counts up to 48000/μL in both this study and the previous ACT KNOW trial [16].

Clinical outcomes with AL and ASMQ were similar despite differences in pharmacokinetic/pharmacodynamic properties between these medications. The proportion of patients with negative microscopy at 24 hours was only 1.1-fold greater in those treated with ASMQ [16] versus AL, and there was no difference in bed occupancy or risk of anemia at follow-up. The different artemisinin derivatives, artemether and artesunate, found in AL and ASMQ respectively, are both metabolized in vivo to the same active component dihydroartemisinin [21] and have been shown to have similar in vitro mean 50% inhibitory concentrations of 0.90 nmol/L for clinical *P. knowlesi* isolates [20]. However, in contrast to ASMQ, AL is administered as a twice-daily dose, with an artemether component of 1.8 mg/kg, compared with the once-daily dosing of ASMQ, with an artesunate component of 4 mg/kg [21]. 

AL is also highly lipophilic, and optimal oral absorption requires coadministration of fatty foods [21]. In routine clinical practice in countries such as Malaysia with mandatory hospital admission for malaria, and also many clinical trials evaluating antimalarial efficacy for *P. falciparum* [35], fatty foods are not regularly coadministered with AL. Despite the presumed reduced oral bioavailability of AL for patients not receiving fatty foods in this study, the recommended weight-based dosing of AL was sufficient for parasite clearance. This is consistent with highly drug-sensitive *P. knowlesi* parasites from zoonotic transmission to humans. Patients in both clinical trials were aparasitemic within 72 hours, and both ACTs are administered for 3 days. Therefore, with the fast 24-hour life-cycle of *P. knowlesi* and high efficacy of the artemisinin component of both ACTs, the shorter half-life of the partner drug for AL, lumefantrine, of about 3 days, did not confer any disadvantage for eliminating any residual parasites compared with mefloquine, with a half-life of about 21 days [21].

Both AL and CQ demonstrated high safety and tolerability, with minimal adverse events. The most commonly reported adverse event was nonsevere rash or itch, seen in 12% of patients treated with AL, with recognized drug-related gastrointestinal disturbance in <5%. AL and CQ were also both tolerated well by the 12 children ≤12 years of age. Suboptimal dosing of AL with children owing to administration as tablets or fractions of tablets in dose-weight bandings [36] had no detectable impact on safety or therapeutic efficacy, although the small number of children in this study limits definitive evaluation. AL did not demonstrate a faster fever clearance than CQ, and although the risk of anemia during follow-up was decreased in those treated with AL compared with CQ, this difference was not statistically significant. 

These findings are in contrast to comparisons of ASMQ and CQ [16], but the evaluation of these secondary outcomes was limited by the smaller sample size in the current study. The low prevalence of *P. knowlesi* gametocytes in about 25% of patients at presentation was probably underestimated by microscopy, with minimum estimates of 85% in a previous study using a sensitive *Pks25* PCR assay [16]. However, the lack of gametocytes at follow-up indicates that both AL and CQ are effective at reducing the sexual stage of *P. knowlesi*, and risk of onward transmission after treatment is minimal, with other transmission-blocking agents, such as primaquine, not required.

In conclusion, our findings support the use of AL as a first-line treatment over CQ for uncomplicated knowlesi malaria, allowing a universal policy of ACT for all species of malaria in this region [24]. Although CQ continues to demonstrate adequate therapeutic efficacy, ACT has a number of advantages related to earlier parasite clearance and hospital discharge and decreased risk of anemia. Furthermore, CQ is not recommended for the treatment of presumed uncomplicated knowlesi malaria owing to the frequent microscopic misidentification of *P. knowlesi* as *P. falciparum* or *P. vivax* [8], both highly CQ-resistant in this region [22, 37]. In addition, faster-acting ACT treatment may minimize the risk of severe complications for *P. knowlesi* infections with parasitemia underestimated by microscopy. AL has some advantage in safety and tolerability over ASMQ because of the small risk of severe mefloquine-related neuropsychiatric sequelae [25], despite otherwise comparable clinical outcomes. 

Results of this and the previous RCT seem generalizable to other areas of Malaysia and Southeast Asia where AL and ASMQ are used as first-line therapies for uncomplicated malaria [21, 24]. Other ACTs, such as dihydroartemisinin-piperaquine, used in coendemic areas of Indonesia [6] for first-line blood-stage treatment of falciparum and vivax malaria, including misidentified *P. knowlesi* infections, are also likely to be efficacious in the treatment of uncomplicated knowlesi malaria, although they require further evaluation.

## Notes

***Author contributions.*** M. J. G., T. W., B. E. B., R. N. P., T. W. Y., and N. M. A. designed the study, and M. J. G., T. W., G. S. R., C. S. W., and T.W.Y. supervised patient enrollment and data collection. M. J. G. did the statistical analysis, with input from N. M. A.; M. J. G. wrote the first manuscript draft, B. E. B., T.W.Y., and N. M. A. edited the initial drafts, and all authors reviewed the final manuscript. M. J. G. had full access to all the data in the study and had final responsibility for the decision to submit for publication.

***Acknowledgments***. We thank the participants in this study and the clinical and laboratory research staff, including Ema Istiana binti Israk, Sitti Saimah binti Sakam, Norfarain binti Mohamed Yassin, Salwah Hamit binti Hamid, Noorazela binti Mohamed Yassin, Areys Benjamin, Wilhelmina Nevir, Siti Norfazaziwantie binti Redzuan, and Maslianah binti Sintom. Sincere appreciation also to the hospital directors at the study sites, the director-general of health, Malaysian Ministry of Health, the head of the Clinical Research Centre, Malaysia, Dr Goh Pik Pin, and colleagues and staff of the UK Medical Research Council Monkeybar Project. We would also like to acknowledge Kim Piera and Ammar Aziz for conducting the PCR species confirmation and other logistical support.

***Disclaimer***. The sponsor of the study had no role in study design, data collection, data analysis, data interpretation, or writing of the report.

***Financial support***. This study was supported by the Malaysian Ministry of Health (grant BP00500420) and the Australian National Health and Medical Research Council (program grant 1037304; project grant 1045156; fellowships 1042072 to N. M. A., 605831 to T. W. Y., and 1088738 to B. E. B.; scholarship 1074795 to M. J. G.; and grant 1131932 [the HOT NORTH initiative]). R. N. P. is a Wellcome Trust senior fellow in clinical science (fellowship 200909).

***Potential conflicts of interest.*** All authors: No reported conflicts of interest. All authors have submitted the ICMJE Form for Disclosure of Potential Conflicts of Interest. Conflicts that the editors consider relevant to the content of the manuscript have been disclosed.

## References

[1] Shearer FM, Huang Z, Weiss DJ Estimating geographical variation in the risk of zoonotic *Plasmodium knowlesi* infection in countries eliminating malaria. PLoS Negl Trop Dis 2016; 10:e0004915. 27494405 10.1371/journal.pntd.0004915 PMC4975412

[2] Singh B, Daneshvar C Human infections and detection of *Plasmodium knowlesi*. Clin Microbiol Rev 2013; 26:165–84. 23554413 10.1128/CMR.00079-12 PMC3623376

[3] William T, Rahman HA, Jelip J Increasing incidence of *Plasmodium knowlesi* malaria following control of *P. falciparum* and *P. vivax* malaria in Sabah, Malaysia. PLoS Negl Trop Dis 2013; 7:e2026. 23359830 10.1371/journal.pntd.0002026 PMC3554533

[4] William T, Jelip J, Menon J Changing epidemiology of malaria in Sabah, Malaysia: increasing incidence of *Plasmodium knowlesi*. Malar J 2014; 13:390. 25272973 10.1186/1475-2875-13-390 PMC4195888

[5] Yusof R, Lau YL, Mahmud R High proportion of knowlesi malaria in recent malaria cases in Malaysia. Malar J 2014; 13:168. 24886266 10.1186/1475-2875-13-168 PMC4016780

[6] Lubis IND, Wijaya H, Lubis M Contribution of *Plasmodium knowlesi* to multispecies human malaria infections in North Sumatera, Indonesia. J Infect Dis 2017; 215:1148–55. 28201638 10.1093/infdis/jix091 PMC5426374

[7] Herdiana H, Cotter C, Coutrier FN Malaria risk factor assessment using active and passive surveillance data from Aceh Besar, Indonesia, a low endemic, malaria elimination setting with *Plasmodium knowlesi*, *Plasmodium vivax*, and *Plasmodium falciparum*. Malar J 2016; 15:468. 27619000 10.1186/s12936-016-1523-z PMC5020529

[8] Barber BE, William T, Grigg MJ, Yeo TW, Anstey NM Limitations of microscopy to differentiate *Plasmodium* species in a region co-endemic for *Plasmodium falciparum*, *Plasmodium vivax* and *Plasmodium knowlesi*. Malar J 2013; 12:8. 23294844 10.1186/1475-2875-12-8 PMC3544591

[9] Lee KS, Cox-Singh J, Singh B Morphological features and differential counts of *Plasmodium knowlesi* parasites in naturally acquired human infections. Malar J 2009; 8:73. 19383118 10.1186/1475-2875-8-73 PMC2676309

[10] William T, Menon J, Rajahram G Severe *Plasmodium knowlesi* malaria in a tertiary care hospital, Sabah, Malaysia. Emerg Infect Dis 2011; 17:1248–55. 21762579 10.3201/eid.1707.101017 PMC3381373

[11] Rajahram GS, Barber BE, William T, Menon J, Anstey NM, Yeo TW Deaths due to *Plasmodium knowlesi* malaria in Sabah, Malaysia: association with reporting as *Plasmodium malariae* and delayed parenteral artesunate. Malar J 2012; 11:284. 22905799 10.1186/1475-2875-11-284 PMC3472242

[12] Rajahram GS, Barber BE, William T Falling *Plasmodium knowlesi* malaria death rate among adults despite rising incidence, Sabah, Malaysia, 2010-2014. Emerg Infect Dis 2016; 22:41–8. 26690736 10.3201/eid2201.151305 PMC4696710

[13] Barber BE, William T, Grigg MJ, Piera K, Yeo TW, Anstey NM Evaluation of the sensitivity of a pLDH-based and an aldolase-based rapid diagnostic test for diagnosis of uncomplicated and severe malaria caused by PCR-confirmed *Plasmodium knowlesi*, *Plasmodium falciparum*, and *Plasmodium vivax*. J Clin Microbiol 2013; 51:1118–23. 23345297 10.1128/JCM.03285-12 PMC3666806

[14] Grigg MJ, William T, Barber BE Combining parasite lactate dehydrogenase-based and histidine-rich protein 2-based rapid tests to improve specificity for diagnosis of malaria Due to *Plasmodium knowlesi* and other *Plasmodium* species in Sabah, Malaysia. J Clin Microbiol 2014; 52:2053–60. 24696029 10.1128/JCM.00181-14 PMC4042751

[15] Foster D, Cox-Singh J, Mohamad DS, Krishna S, Chin PP, Singh B Evaluation of three rapid diagnostic tests for the detection of human infections with *Plasmodium knowlesi*. Malar J 2014; 13:60. 24548805 10.1186/1475-2875-13-60 PMC3931291

[16] Grigg MJ, William T, Menon J Artesunate-mefloquine versus chloroquine for treatment of uncomplicated *Plasmodium knowlesi* malaria in Malaysia (ACT KNOW): an open-label, randomised controlled trial. Lancet Infect Dis 2016; 16:180–8. 26603174 10.1016/S1473-3099(15)00415-6 PMC4753673

[17] Barber BE, William T, Grigg MJ A prospective comparative study of knowlesi, falciparum, and vivax malaria in Sabah, Malaysia: high proportion with severe disease from *Plasmodium knowlesi* and *Plasmodium vivax* but no mortality with early referral and artesunate therapy. Clin Infect Dis 2013; 56:383–97. 23087389 10.1093/cid/cis902

[18] Daneshvar C, Davis TM, Cox-Singh J Clinical and parasitological response to oral chloroquine and primaquine in uncomplicated human *Plasmodium knowlesi* infections. Malar J 2010; 9:238. 20723228 10.1186/1475-2875-9-238 PMC2933701

[19] Barber BE, Grigg MJ, William T, Yeo TW, Anstey NM The treatment of *Plasmodium knowlesi* malaria. Trends Parasitol 2017; 33:242–53. 27707609 10.1016/j.pt.2016.09.002

[20] Fatih FA, Staines HM, Siner A Susceptibility of human *Plasmodium knowlesi* infections to anti-malarials. Malar J 2013; 12:425. 24245918 10.1186/1475-2875-12-425 PMC3874596

[21] World Health Organization. Guidelines for the treatment of malaria. 3rd ed Geneva, Switzerland: World Health Organization; 2015.

[22] Grigg MJ, William T, Menon J Efficacy of artesunate-mefloquine for chloroquine-resistant *Plasmodium vivax* malaria in Malaysia: an open-label, randomized, controlled trial. Clin Infect Dis 2016; 62:1403–11. 27107287 10.1093/cid/ciw121 PMC4872287

[23] World Health Organization. World malaria report 2016 Geneva, Switzerland: WHO, 2016 Available at: http://apps.who.int/iris/bitstream/10665/252038/1/9789241511711-eng.pdf?ua=1. Accessed 14 December 2016.

[24] Ministry of Health Malaysia Vector Borne Disease Control Program (VBDCP). Management guidelines of malaria in Malaysia Kuala Lumpur, Malaysia: Ministry of Health; 2013 Available at: http://www.moh.gov.my/index.php. Accessed 18 June 2014.

[25] Lee SJ, Kuile ter FO, Price RN, Luxemburger C, Nosten F Adverse effects of mefloquine for the treatment of uncomplicated malaria in Thailand: a pooled analysis of 19, 850 individual patients. PLoS One 2017; 12:e0168780. 28192434 10.1371/journal.pone.0168780 PMC5305067

[26] Barber BE, William T, Dhararaj P Epidemiology of *Plasmodium knowlesi* malaria in north-east Sabah, Malaysia: family clusters and wide age distribution. Malar J 2012; 11:401. 23216947 10.1186/1475-2875-11-401 PMC3528466

[27] Grigg MJ, Cox J, William T Individual-level factors associated with the risk of acquiring human *Plasmodium knowlesi* malaria in Malaysia: a case-control study. Lancet Planet Health 2017; 1:e97–e104. 28758162 10.1016/S2542-5196(17)30031-1 PMC5531251

[28] World Health Organization. Severe malaria. Trop Med Int Health 2014; 19(suppl 1):7–131. 25214480 10.1111/tmi.12313_2

[29] WorldWide Antimalarial Resistance Network (WWARN). Methodology for the WWARN parasite clearance estimator 2015 Available at: http://www.wwarn.org/tools-resources/pce-methodology. Accessed 5 February 2015.

[30] World Health Organization. Haemoglobin concentrations for the diagnosis of anaemia and assessment of severity 2011 Available at: http://www.who.int/vmnis/indicators/haemoglobin.pdf. Accessed 10 June 2015.

[31] Imwong M, Tanomsing N, Pukrittayakamee S, Day NP, White NJ, Snounou G Spurious amplification of a *Plasmodium vivax* small-subunit RNA gene by use of primers currently used to detect *P. knowlesi*. J Clin Microbiol 2009; 47:4173–5. 19812279 10.1128/JCM.00811-09 PMC2786678

[32] Padley D, Moody AH, Chiodini PL, Saldanha J Use of a rapid, single-round, multiplex PCR to detect malarial parasites and identify the species present. Ann Trop Med Parasitol 2003; 97:131–7. 12803868 10.1179/000349803125002977

[33] Daneshvar C, Davis TM, Cox-Singh J Clinical and laboratory features of human *Plasmodium knowlesi* infection. Clin Infect Dis 2009; 49:852–60. 19635025 10.1086/605439 PMC2843824

[34] Barber BE, Grigg MJ, William T Effects of aging on parasite biomass, inflammation, endothelial activation, microvascular dysfunction and disease severity in *Plasmodium knowlesi* and *Plasmodium falciparum* malaria. J Infect Dis 2017; 215:1908–17. 28863470 10.1093/infdis/jix193 PMC8453637

[35] WorldWide Antimalarial Resistance Network (WWARN). Artemether-lumefantrine treatment of uncomplicated *Plasmodium falciparum* malaria: a systematic review and meta-analysis of day 7 lumefantrine concentrations and therapeutic response using individual patient data. BMC Med 2015; 13:227. 26381375 10.1186/s12916-015-0456-7 PMC4574542

[36] WorldWide Antimalarial Resistance Network (WWARN). The effect of dose on the antimalarial efficacy of artemether-lumefantrine: a systematic review and pooled analysis of individual patient data. Lancet Infect Dis 2015; 15:1–11. 25788162 10.1016/S1473-3099(15)70024-1 PMC4632191

[37] Hakim SL, Roohi SS, Zurkurnai Y *Plasmodium falciparum*: increased proportion of severe resistance (RII and RIII) to chloroquine and high rate of resistance to sulfadoxine-pyrimethamine in Peninsular Malaysia after two decades. Trans R Soc Trop Med Hyg 1996; 90:294–7. 8758083 10.1016/s0035-9203(96)90258-8

